# Adolescent self-harm in Ghana: a qualitative interview-based study of first-hand accounts

**DOI:** 10.1186/s12888-020-02599-9

**Published:** 2020-06-01

**Authors:** Emmanuel N-B Quarshie, Mitch G. Waterman, Allan O. House

**Affiliations:** 1 Department of Psychology, University of Ghana, P.O. Box LG 84, Legon, Accra, Ghana; 2 School of Psychology, University of Leeds, Leeds, UK; 3 Leeds Institute of Health Sciences, University of Leeds, Leeds, UK

**Keywords:** Adolescents, Adultification, Ghana, Self-harm, Street-connected adolescents, Sub-Saharan Africa, Suicide, Suicide attempt, Tramadol

## Abstract

**Background:**

Recent prevalence studies suggest that self-harm among adolescents in sub-Saharan Africa is as common as it is in high income countries. However, very few qualitative studies exploring first-person accounts of adolescent self-harm are available from sub-Saharan Africa. We sought to explore the experiences and first-person perspectives of Ghanaian adolescents reporting self-harm - for deeper reflections on the interpretive repertoires available in their cultural context for making sense of self-harm in adolescents.

**Methods:**

Guided by a semi-structured interview protocol, we interviewed one-to-one 36 adolescents (24 in-school adolescents and 12 street-connected adolescents) on their experiences of self-harm. We applied experiential thematic analysis to the data.

**Results:**

Adolescents’ description of the background to their self-harm identified *powerlessness* in the family context and unwanted *adultification* in the family as key factors leading up to self-harm among both in-school and street-connected adolescents. Adolescents’ explanatory accounts identified the contradictory role of adultification as a protective factor against self-harm among street-connected adolescents. Self-harm among in-school adolescents was identified as a means of “enactment of tabooed emotions and contestations”, as a “selfish act and social injury”, as “religious transgression”, while it was also seen as improving social relations.

**Conclusions:**

The first-person accounts of adolescents in this study implicate familial relational problems and interpersonal difficulties as proximally leading to self-harm in adolescents. Self-harm in adolescents is interpreted as an understandable response, and as a strong communicative signal in response to powerlessness and family relationship difficulties. These findings need to be taken into consideration in the planning of services in Ghana and are likely to be generalisable to many other countries in sub-Saharan Africa.

## Background

According to the World Health Organisation (WHO), self-harm is “an act with non-fatal outcome in which an individual deliberately initiates a non-habitual behaviour, that without intervention from others will cause self-harm, or deliberately ingests a substance in excess of the prescribed or generally recognised therapeutic dosage, and which is aimed at realising changes that the person desires via the actual or expected physical consequences” [1, 2].

Self-harm among young people is a recognised problem in the mental health of populations in high income countries, where it is associated with a number of poor outcomes including eventual suicide [3, 4]. Although much of our understanding comes from research in the UK, North America, and Australia, [5–7] regional reviews have suggested it is also a common problem in low income countries [8–11].

In addition to continuing uncertainty about the exact prevalence of self-harm, we know little about the meaning of self-harm in the African context [12, 13]. An exploration of meanings is best undertaken using qualitative research, which allows for a more open-ended exploratory approach that can identify unexpected and highly personal responses from individuals [14–17]. Under the circumstances it is disappointing that we have found few qualitative studies exploring first-person accounts of self-harm from sub-Saharan Africa, and most that we did identify have been conducted in South Africa [18–21]. We found only one extant clinical case study from Ghana, involving an adolescent female patient who reported failed sense of autonomy in her family, and family harassment and dispute as the main reasons for her self-harm [22].

To the best of our knowledge, the present study represents the first effort at providing first-person accounts from Ghana of the meanings of self-harm as held by a non-clinical sample of adolescents with a self-harm history. Such research is needed in African countries to explore the personal thoughts, feelings, motivations, experience of life events and their socio-cultural context, as young people describe and explain their pathways to self-harm [12].

### Aims and objectives

We sought first-person accounts from Ghanaian adolescents with personal experience of self-harm, of the circumstances leading up to the act, and of the reasons they offered for self-harm as a response to those circumstances. Our specific research questions were: (a) What are the adolescents’ accounts of the circumstances leading up to their self-harm? (b) How do the adolescents make sense of their self-harm as a response to their circumstances?

## Methods

### Design and setting

A qualitative cross-sectional observational study involving one-to-one semi-structured interviews with in-school adolescents and street-connected adolescents in the Greater Accra region of Ghana [23]. The interviews took place between August 2017 and April 2018.

### Sample

In a previous questionnaire survey, we asked adolescents who reported a history of self-harm to sign an additional consent form expressing their interest to participate in the interview study [24]. In all, 36 adolescents (24 in-school adolescents and 12 street-connected adolescents) signed the form and were recruited. Guided by the information power model for obtaining adequate sample size for qualitative studies, [25] we sought enough participants to be able to take account of the substantial heterogeneity of experience in the population of interest, especially in relation to membership in each of the two main sub-groups.

We did not undertake a medical or psychiatric assessment of the participants, beyond noting that all our interviewees had mental capacity and were able to give fully informed consent to participation. Thus, as recommended by Aptekar and Stoecklin, [26] we performed a mental status exam of potential participants by assessing the mental and physical health state of the participants through observation at the time of interview; looking out for visible signs indicative of ill health, neurological impairment or signs of alcohol or drug intoxication or withdrawal.

The in-school adolescents (18F, 6 M) were aged between 15 and 20 years (mean = 17 years). They reported families of sibling size between 1 and 8 (mean = 4). Most self-identified as heterosexual, with two bisexual and one transgender. Three students self-identified as Muslim and 21 as Christians. Reported lifetime self-harm frequency ranged between 1 and 7 (mean = 2). Thirteen (54%) indicated that prior to their own self-harm they knew someone (directly or indirectly) who had self-harmed or died by suicide in their family, community, school, or among their friends. Similarly, 88% (*n* = 21) reported that they had seen self-harm scenes in television content prior to their own self-harm.

The 12 street-connected adolescents (8F, 4 M) were aged between 13 and 19 years (mean 16 years). They reported that they had been in the street situation between one and 3 years (Mean = 1.8). Two participants indicated that they had a bisexual orientation, two were unemployed and two were still attending school. Their families of origin had sibling size varying between 1 and 8 (mean 5); all but two reported their fathers had more than one wife. The street-connected adolescents reported lifetime self-harm frequency varying between 1 and 2 (mean = 1.4). Nine indicated that prior to their own self-harm, they knew someone (directly or indirectly) who had self-harmed or died by suicide in their family, community, school, or among their friends. Four mentioned that prior to their first episode of self-harm, they had seen self-harm content on television.

### Interview

The semi-structured interview was made up of two parts. The first was a narrative part where each participant was asked to provide a description of their experience of self-harm and general view about self-harm in Ghana. The second part of the interview (problem-focused) involved questions probing relevant details that were not sufficiently addressed in the responses to the open questions. The development of the problem-focused part was guided by the antecedent-behaviour-consequence framework for reviewing and assessing self-harm in adolescents [27]. Care was taken to ensure that the participants’ understanding of self-harm excluded unintentional injury or harm resulting from accidents, and was not limited to self-injurious or self-poisoning with suicidal motives (attempted suicide) [28]. The language for each interview (English, Ga or Twi) was based upon the participant’s choice.

All the interviews were audio recorded and transcribed by EQ, who undertook all the interviews and made field notes during and immediately after each interview to provide context for the transcripts. EQ is a Ghanaian and a trained Community Psychologist – with specialisation in child and adolescent mental health – who has several years of experience in community and mental health promotion and research in Ghana. EQ’s ‘insider’ status facilitated the establishment of rapport and enhanced the capacity for empathy in conducting the interviews [29, 30]. The interviews, which were conducted 1:1 with nobody else present, took place within private but secure spaces such as, nearby clinic consulting rooms, offices of community centres, charity facilities, university research offices, designated unoccupied classrooms, and quiet corners of restaurants. Interviews with the in-school adolescents lasted longer (between 60 and 90 min) than the interviews with the street-connected adolescents (between 23 min and 35 min).

### Analysis

The transcripts of the interviews conducted entirely in Ga or the Twi language and in a mix of English with Ga or Twi were reviewed by two experts in Ghana: a qualitative researcher with interest in adolescent mental health issues and a linguistics researcher. Consensus reached by the experts during the review sessions helped in producing precise and meaningful translations of the language and terms, including figurative expressions used by the participants. Where a statement or term did not readily translate into English, it was maintained in its original form alongside a literal English translation.

After each day’s session of interviews, the audio-recorded interviews were immediately transferred from the audio recorder onto a university password-protected storage system. Transcripts of the interviews were kept separately in a locked filling cabinet in a university research office; keys and access to the filling cabinet were limited to only the authors. Only the anonymised copies of the transcripts were kept and used for analysis.

Experiential thematic analysis was used by the three authors to analyse the transcribed interviews [31]. This technique is a variant of thematic analysis that focuses on the participants’ standpoint – how participants experience and make sense of their world [31]. We followed a 6-phase approach to analyse the data [32, 33]: familiarising ourselves with the data, generating initial codes, searching for themes, reviewing themes, defining and naming themes, and producing the report. Emergent themes were discussed between all three authors until consensus was reached. To preserve the reliability and validity of our findings further, critical comments from the co-authors (who are international researchers but ‘outsiders’ to the Ghanaian culture) ensured that the first author’s personal biases and assumptions, as an ‘insider’ of the socio-cultural context of Ghana, did not blur the interpretation of the data and equally important context relevant factors were not overlooked in the analysis and interpretation of the data [29, 30]. Because of the nature of the sample – the sensitivity of self-harm in Ghanaian society and the reality of street-living in Accra – it was not possible to return to the participants to validate our findings.

## Results

We organised the findings around two main themes each with their subthemes: adolescents describe the background to self-harm, and adolescents explain their self-harm (Table 1 presents summary of the themes).

**Table 1:** Summary of themes

Thematic area	Adolescent group	Theme	Brief explanation
1) Adolescents describe the background to self-harm	In-school	i. Powerlessness related to age and gender	▪ Lack of power among young people and girls to take personal decisions and to contribute to or influence decisions and roles in the family.
		ii. Parental modelling and parent-child incongruent expectations	▪ Conflicting role expectations between parents and their adolescent children.
		iii. Parental criticism	▪ Criticism of adolescents by their parents.
		iv. Perceived unfair application of the rule of punishment	▪ Perception that the rules governing punishment of deviant behaviours were unfairly or inconsistently applied by significant other adults (e.g., parents, older siblings etc.), leading to feeling unloved and uncared for.
		v. Perceived family mistrust and betrayal	▪ False accusations and rumours where adolescents did not receive support and defence from their family.
		vi. Early adultification	▪ The practice of making a child act as a primary caregiver providing emotional, material or instrumental support to adult relatives, younger siblings or to self, before the child is emotionally prepared to do so.
		vii. Diabolical control	▪ Manipulations by unseen evil forces.
	Street-connected	i. Self-harm as a response to adultification in family of origin	▪ The practice of making a child act as a primary caregiver providing emotional, material or instrumental support to adult relatives, younger siblings or to self, before the child is emotionally prepared to do so.
		ii. Self-harm as a response to acculturative stress of street living	▪ For newcomers to street living, self-harm was a response to the mental strain experienced in adapting to the harsh realities of street living.
		iii. Self-harm as a response to conflict of conduct norms in charity facility	▪ For newcomers to charity facilities, self-harm was in response to the behavioural difficulty experienced in adapting to the controlled culture of charity facilities.
2) Adolescents explain their self-harm	In-school: meaning-making	i. Enactment of tabooed emotions and contestations	▪ A means of contesting or protesting unbearable scolding, criticism, and (perceived) abuse by their parents.
		ii. Avenge excessive control and punishment by parents	▪ A means of avenging and ending the excessive control and punishment by their parents, particularly, fathers.
		iii. Responding to and management of negative emotions and circumstances	▪ A way of managing acute negative emotions or as a response to emotional disturbance or negative (interpersonal) circumstances.
	In-school: consequences and influence on recovery	i. Self-harm as selfish act and social injury	▪ Self-harm was personally helpful but socially injurious to significant others; this motivated stopping self-harm.
		ii. Self-harm improves social relations	▪ Self-harm led to improvement in social relationships with significant other adults; this motivated stopping self-harm.
		iii. Self-harm worsens abuse	▪ Harsh punishment or abuse linked to self-harm worsened, particularly, where the self-harm was discovered because it resulted in hospitalisation.
		iv. Self-harm as religious transgression	▪ A breach of religious tenets; this motivated stopping self-harm.
	Street-connected: explanatory accounts	i. Reliance on peer and surrogate family support	▪ Adolescents relied on surrogate families and friends for financial and emotional support and protection; this motivated stopping self-harm.
		ii. Reliance on charity support	▪ Support obtained from attending charity facility helped street-connected adolescents in stopping or avoiding self-harm
		iii. Early adultification in the streets	▪ Taking on the role of an adult to care and provide for one’s own needs motivated stopping self-harm.
		iv. Use of substitutes	▪ Adolescents pursued (usually, harmful) alternative acts to distract themselves from self-harm (e.g., substance use).

### Adolescents describe the background to self-harm

#### “The child is always wrong”: interviews with in-school adolescents

We identified seven themes related to the idea of children’s powerlessness as reported by the in-school adolescents. Six themes attributed it to adult authority: (i) “powerlessness related to age and gender”, (ii) “parental modelling and parent-child incongruent expectations”, (iii) “parental criticism”, (iv) “perceived unfair application of the rule of punishment”, (v) “perceived family mistrust and betrayal”, and (vi) “early adultification”. One theme related powerlessness to, (vii) diabolical control.

##### Powerlessness related to age and gender

In the African conceptual scheme young people are generally considered as inexperienced in life, requiring constant adult supervision and guidance [34]. Thus, the socio-cultural lore within the Ghanaian family remains that the child is always wrong [35, 36].

*There are times when an older person can be totally wrong, and the child can be right. It is good for an older person to admit his or her mistake in front of the child [ …] or apologise to the child. The mentality that an older person is always right, but the child is wrong is very bad. Whenever my mom behaves that way, it hurts me, I feel as if I am a bad boy, and that’s what made me cut myself in the first place, because you feel as if there is nothing good you can do (Chris, male, 18 years).*

For female adolescents, the sense of powerlessness is related to both their young age and gender.

*I’m the first girl in the house, so I’m supposed to cook. I cook, I wash [hand-wash clothes] and I sweep… Occasionally, he [my father] drags us [the children] into his fight and ‘charade’ with my mother in the house. He hits and slaps me because I question him [about] why he doesn’t talk to anyone in the house and I do it in front of him openly. He says I’m a girl and I can’t question his behaviour. He says it’s because my mother is not training me properly. But how come that my brothers, [um…] even my younger brother can ask you questions but I cannot ask you [my father] questions because I am a girl? I can’t ask you to change your bad behaviour? Like, I mean, it’s weird.* (Sheila, female, 16 years).

##### “… We are in the 21st century…who does that?”: parental modelling and parent-child incongruent expectations

This theme relates to the conflicting role expectations between parents and their adolescent children. Some of the adolescents reflected that their parents had modelled certain strict traditional parental roles from their own parents which they (the adolescents) found to be incongruent with contemporary parent-child relationship expectations. Particularly, the adolescents identified their parents’ traditional roles related to controlling adolescent social relationships, corporal punishment of adolescents, parental supervision and monitoring of adolescents, and everyday family interactions to be incongruent with contemporary culture.

##### “…she [my mother] called me ‘kojo besia’”: parental criticism

Respondents described situations in their families where their parents criticised them for behaving in ways that were inconsistent with traditional age and gender role ideologies and expectations. They reported feelings of frustration, humiliation and anger because their conduct was continuously criticised.

*For several times and days, she [my mother] called me ‘kojo besia’. My eyes were teary, and I was angry ‘cos I was feeling bad, but I tried to control myself. She [my mother] was, like, ‘anytime you are going to wash [hand-wash clothes], you will be delaying. Instead of you to wash quickly you will be taking hours’. [um…] but that’s not true, I don’t waste time washing. At first, I used to wash only my clothes and I finished within a few minutes, but these days I wash her clothes, her husband’s [my stepfather’s] and those of my younger stepbrothers combined, plus mine too. I’m a boy, she [my mother] is supposed to wash, not me. But I wash, and you call me kojo besia. [um…] I’m not a girl and I’m not lazy (Chris, male, 18 years).*

In Ghana, kojo besia literally means a child born on a Monday (Kojo) who is a girl (besia). Kojo besia is used metaphorically to mean a male who behaves in ways that are deemed stereotypical of females [37]. Chris might have found the descriptive, Kojo besia, not only pejorative but also as a label which sought to place him outside the traditional male gender role and masculinity ideals such as hard work and strength.

##### “I wonder why… they don’t find anything wrong with that”: perceived unfair application of the rule of punishment

The socialisation of children and young people in the Ghanaian family is characterised by cultural sanctions: acceptable behaviours are rewarded whereas deviation from norms of socially acceptable behaviour is punished [38]. For some of the participants in this study, the decision to self-harm was also informed by their perception that the rules governing punishment of deviant behaviours were unfairly or inconsistently applied, giving them feelings that they were unloved and uncared for by their parents. For example,

Abbie had been hospitalised for self-harm following physical punishment by her parents and older brother, based on reports that she had been found to be interacting with Joe (a young man in their neighbourhood), which suggested that she was romantically involved with Joe. She maintained that the reports were unsubstantiated and false. According to Abbie, however, she had not been punished at all for interacting with Malik (another young man in the neighbourhood), who was even closer and spent more time with her than Joe. In her view the same “offence” has been punished in one instance but remains unpunished in another instance.

##### “I expect my mother to defend me”: perceived family mistrust and betrayal

Especially the female participants reported that they felt mistrusted and betrayed by their families. The cases of perceived family mistrust and betrayal were particularly related to instances where the female adolescents maintained their innocence of accusations and rumours related to engaging in premarital romantic activities or sexual relationships.

*I expect my mother to defend me because she knows me well, whether I go out or not, she knows it all because I live in the same house with her and we share the same bedroom. I always blame her […] I felt better dead, the pain was too much […] like, you have not done something, and you are accused of doing it, it’s painful. They see me like a bad girl, but I’m not […] it beats my imagination why my mother cannot defend me, because I’m the only person who helps her (Abbie, female, 16 years).*

##### “I’m only a small boy, why should I be struggling as if I am a father?” early adultification

Adultification happens when a child or an adolescent is forced to assume adult roles of acting as a primary caregiver providing emotional, material or instrumental support to adult relatives, younger siblings or themselves, before they are emotionally prepared to do so [39, 40]. In the present study, comparatively, more male participants reported that they were “adultified” at younger ages and they linked their self-harm to the motivation to end the struggles that came with their adultification.

In most of the instances, the participants came from broken families where they had witnessed parental separation, their parents were divorced, one parent was deceased or had ill health, or the adolescent and their siblings were being looked after by a single parent or a grandparent. These circumstances forced the participants to take on adult roles as primary caregivers by working to provide for their personal material needs (e.g., food, school fees, etc.) and those of their siblings and the financial needs of other adult relatives such as parents or grandparents.

*[…] my mother became very ill, she was always in bed, and she could not work anymore. So, I had to be working to feed myself, pay my school fees, and to give her something to also take care of herself […] I was in a private school, but I stopped because of the school fees, I couldn’t pay, so I moved to a government school […] I did any work at all, I was weeding people’s houses, working as a trotro mate,* 1*carrying concrete at construction sites, and many more […] Yeah, it was very difficult, sometimes because of just food to eat for the day, I had to go and work, work, work before I could get money to buy food to eat […] I was suffering and struggling at that young age, while my friends were not doing anything like that. I was suffering too much (Steve, male, 18 years).*

##### “…it was the work of the devil…”: diabolical control

Some participants attributed their self-harm to manipulations by unseen evil forces. They reflected that they had lost self-control and acted carelessly in the heat of the moment leading up to their self-harm.

*[…] The whole [self-harm] thing happened so quickly; I drank the medicines without thinking. But I think, at that moment it was the devil at work. Yeah, some of these self-harm behaviours can be caused by evil spirits […] and we need the pastors to intercede with serious prayers for us young people (Bob, male, 15 years).*

#### “If I wanted to live here, then I would have to be strong”: interviews with street-connected adolescents

Four of the 12 street-connected adolescents reported that their first and only lifetime episode of self-harm happened while living with their original families, before coming into the street living situation, whereas eight reported that they self-harmed while living on the streets. The street-connected adolescents who self-harmed while living with their original families did so as a response to “adultification in their families of origin”, whereas those who self-harmed while in the street situation did so as a response to the “acculturative stress of street living”, and the “conflict of conduct norms in the charity facilities” they attended.

##### Self-harm as a response to adultification in family of origin

Four adolescents reported that their basic needs, particularly, food and education were not provided for while living with their families. However, each mentioned that they were made to work as adults, providing material and instrumental support to their adult relatives, to the neglect of their own personal needs:

*Before my mother died, I was taking care of her for the whole time when she was ill. By then, I was only 15 years old; I dropped out of school. Every day, I bathed her, fed her, and changed her clothes and other things […] My older siblings came around, but they didn’t care if all was well with me […] Things were difficult, sometimes even food to eat was difficult to get. My grandmother was not working. I did not know what to do. I felt nobody in this world liked me, no one loved me or cared about me, so I wanted to die […] (Maud, female, 19 years).*

##### Self-harm as a response to acculturative stress of street living

Initial negative experiences of the realities of the street culture made the young people hopeless and powerless.

*[…] When I came here (I mean Accra) for the first time, things were difficult, usually no food to eat, no money to buy food, no work to do, nothing. Sometimes I could be walking in the market and someone would just scream “julɔ eei!” [thief!], when I had not even touched anything or anybody, then people from nowhere would just pounce on me and beat me up, because no one knew me here, I was new. Luckily, after a few days, I began selling polythene bags, but the man I used to work for accused me of stealing his money, but I did not do it. He beat me up and sacked me from his business. That day, I had worked for long hours and made good money for him, but he didn’t pay me my commission for the day. That night, thieves attacked me and made away with my little savings. They had knives and so I couldn’t struggle with them, they could have hurt me or even killed me or something like that […] I felt there was no need to continue living […] (Abdul, male, 16 years).*

Abdul indicated that he learnt a motto from his 14-year old friend that, in order to survive in Accra, “esa ni ohiɛ awa, kaaha ni moko shishiu bo yɛ biɛ” [you have to be strong, don’t let anyone cheat you here].

A female participant also shared her new experience of the street situation which she linked to her self-harm, as follows,

*I had been here [Accra] with my friends for just two weeks […] He said he could show me where I could get more customers, so I went with him. But when we were coming back he made some calls and he made us pass by where his friends lived […] They gave me Malta Guinness, not knowing they had put medicine in it, and that made me sleepy […] All of them slept with me […] It was two of my friends who later came to find me there in pain and brought me here [where we live] […] I felt bad, I felt empty. I tried to swallow plenty of tablets so that I would die, but my cousin (the one who brought us to Accra) told me that if I wanted to live here [in Accra] then I would have to be strong, stay out of trouble with boys I don’t know, and stick with the girls all the time and never walk alone […] That statement my cousin made is what has kept me safe to today (Efia, female, 16 years).*

##### Self-harm as a response to conflict of conduct norms in charity facility

Some street-connected adolescents who attend charity facilities may initially find it distressing when they are not allowed to conduct themselves in certain ways deemed inappropriate by the charity facilities. One of the participants attributed his self-harm to the distress resulting from the conflict of conduct norms he experienced when he initially attended a charity facility.

*When I started coming here [charity facility] at first, if someone troubled me and we started a fight, the [social] workers here would come and separate us and tell us to stop the fight. But whenever it happened that way, I felt cheated and angry because I was not allowed the chance to fight the person or beat the person as I wanted to. So, in the anger I hit my head several times to the wall […] You can meet the person somewhere for another fight, but he can come and report you here [charity facility] the next day, and you will be stopped from coming here [charity facility] again (Edem, male, 14 years).*

### Adolescents explain their self-harm

#### In-school adolescents’ meaning-making

##### “I wanted him to see that I hated what he was doing to me”: enactment of tabooed emotions and contestations

The adolescents, particularly females, self-harmed as a means of contesting or protesting unbearable scolding, criticism, and (perceived) abuse by their parents. Generally, the Ghanaian culture forbids children and young people, even if they are right, from expressing negative emotions (e.g., anger, rage) towards their parents or elders [34, 35]; likewise, it is tabooed to (openly or privately) contest their parents’ or elders’ position on an issue – they are to “submit to parental control, advice, or authority” [34]. Young people who show insubordination or defy this norm of respect and obedience are punished, often by scolding or physical beatings [38]. The rationale for this taboo is to avoid any loss of face and to keep intact the honour and dignity of parents and elderly persons in the family.

*One afternoon when he [my stepdad] called me to his bedroom to have sex with me, I refused, and I ran out of the house; I returned home in the evening. That night, he beat me for not wanting to have sex with him, and he had sex with me by force […] I didn’t want him to sleep with me again, so I drank a [small] bottle of ‘parazone’ [bleach]. I wanted him to see that I hated what he was doing to me […] (Amina, female, 18 years).*

##### “…he’ll probably be arrested and locked away”: avenge excessive control and punishment by parents

For some participants (mostly females), self-harm was a means of avenging and ending the excessive control and punishment by their parents, particularly their fathers.

*Sometimes when he [my father] gets angry, he is beating everybody up, and taking out his frustration on all of us, especially me. While in the kitchen, I look at the fire and I wonder if I burn myself and call the police that he did it, he’ll probably be arrested and locked away. I normally thought if someone would ask me why your hand is burnt, I can say he did it. And I’m sure my mom will not defend him, neither will my siblings (Sheila, female, 16 years).*

##### Responding to and management of negative emotions and circumstances

Some participants reported that they self-harmed as a way of managing acute negative emotions or as a response to emotional disturbance or negative (interpersonal) circumstances.

*“…and one time he hit me, but I didn’t want to cry. So, I just walked to the kitchen, lit up the stove and I put my hand in the fire and I realised that the pain from the burnt stopped me from feeling sad and teary. It’s like psychology – if you’re crying because you are sad, and someone slaps you, you don’t feel the sadness anymore, because the slap takes your attention”. (Sheila, female, 16 years).*

##### Consequences and influence on recovery

Here, the adolescents evaluated their self-harm in terms of its consequences and how the consequences influenced their recovery. Four subthemes emerged to describe this main theme: “self-harm as selfish act and social injury”, “self-harm improves social relations”, “self-harm worsens abuse”, and “self-harm as religious transgression”.

##### Self-harm as selfish act and social injury

With the benefit of hindsight, some of the adolescents evaluated their self-harm as an act of selfishness, which (could have) injured significant others. Self-harm was personally helpful but socially injurious to significant others.

*My mom couldn’t take it that I wanted to kill myself. I think she was so hurt, and as for my twin sister, it was worse, ‘cos she cried and cried […] So, like, you can harm yourself alone, but it affects many people around you, yeah. It’s [self-harm is] a bad behaviour (Julia, female, 17 years).*

##### Self-harm improves social relations

Some of the participants recalled that their self-harm led to improvement in their social relationships with significant other adults – typically between the adolescent and a significant other adult, who was not the perpetrator of the harsh punishment or abuse linked originally to the self-harm.

##### Self-harm worsens abuse

Most of the participants who linked their self-harm to harsh punishment or abuse reported that their self-harm led to immediate relief of their emotional pain; however, the harsh punishment or abuse linked to the self-harm worsened afterwards, particularly where the self-harm was discovered because it resulted in hospitalisation. This response was partly explained by the general collectivistic orientation of the Ghanaian society; in this sense, the obligation to preserve family honour and image overrides individual interests at all times [34, 36]. Young people are expected to put their family’s honour first, before personal needs and interests. Second, attempted suicide and suicide are morally proscribed and legally criminalised in the Ghanaian society [41–43], making self-harm a socially injurious act for the family [44–46]. Finally, it is possible that the unplanned cost of hospitalisation might have also contributed to family tension.

##### Self-harm as religious transgression

For some adolescents, their self-harm amounted to a religious transgression, a judgement which made them remorseful with a resolve not to repeat self-harm in the future.

*Anytime I remember what I did, like, if I picture cutting myself, I feel so bad and guilty, like, I regret it. It’s just so wrong, and I always pray for forgiveness from God, “cos it’s a sin to, like, engage in self-harm (Morris, 20 years).*

Ghanaians tend to view suicidal behaviours within a moral and religious lens [47, 48], what was not clear from the views of the present participants is whether they were aware of these moral and religious tenets against self-harm prior to their own self-harm.

##### Explanatory accounts from street-connected adolescents

Although the street-connected adolescents were able to describe background factors linked to self-harm, they differed from the in-school adolescents in that they offered no explanations for why self-harm might follow such experiences. Instead they reflected upon the perceived factors facilitating recovery or helping them to stop self-harm. Most of the street-connected adolescents (*n* = 7) reported that they had self-harmed only once in their lifetime, whereas five participants reported that they had repeated self-harm at least once. Notably, both repeaters and non-repeaters evaluated their self-harm as unhelpful and a potentially dangerous choice, although some reported that they had obtained immediate emotional release as a result. They indicated that they relied on some sources of support and personal strengths to stop or avoid repeating self-harm: “reliance on peer and surrogate family support”, “reliance on charity support”, “early adultification in the streets”, and “use of unhealthy substitutes”.

“…I can borrow money from my friends…”: reliance on peer and surrogate family support

Many of the adolescents (*n* = 8) reported that they relied on the surrogate family and friends they had on the street for financial and emotional support and protection. Abdul (male, 16 years) said,

*[mm…] there were times I thought about it, maybe to do something bad to myself, when I had no money to buy food and things were hard. But I didn’t actually do it. I don’t harm myself because now I can borrow money from my friends and pay back later or maybe I can go to help some of my friends with their work and when we finish, I get some money for myself, you understand? So, as for self-harm, no, I have not done that again. The thoughts of it come into my mind when things are very hard, but because I help my friends when I have, they also help me when I need help (Abdul, male, 16 years).*

##### Reliance on charity support

All the participants in this study knew about and had positive regard for the charity facilities that provide support for street-connected children and young people within the Greater Accra region. Among other things, the charity facilities provide free drop-in social and educational events, meals, and recreational space for these young people, but more importantly (through the employed services of trained social workers, and in some case psychologists), they organise street-child outreach programmes, street-baby care programmes, and provide temporary shelter and short-term vocational skills training for street-connected children and young people [49]. The participants in this study, particularly, those who were interviewed for this study at the charity facilities indicated that the support that they obtained from attending the charity facility helped them in stopping or avoiding self-harm.

##### “I was my own mother and father”: early adultification in the streets

Some of the adolescents, specifically, those who migrated from other regions to Accra, mentioned that upon reaching the streets of Accra, they realised early on that they were on their own; they were their own parents and therefore there was the need to take on the role of an adult to care and provide for their own needs. Two participants reported that they needed to survive in order to take care of themselves; one adolescent (Maud, female, 19 years) indicated that she had the simultaneous responsibility of working to provide for her own personal needs and those of her child’s survival in the street situation; three adolescents indicated that they work to provide for their personal needs and the needs of their family dependents back in their home towns and regions.

*[yeah…] At first when I came here [Accra] my friends used to help me with money to buy food, but after a few days when I started getting my own money from the kayayei* 2*work, they stopped giving me the money and free things […] Everybody contributed to the food we cooked and we all paid for the place where we slept. At that point, I realised that I was on my own, I was my own mother and father […] So sometimes even if you are sick and the sickness is not serious, you still have to work else you’d go hungry. Doing your own work to take care of yourself makes you strong and keeps your mind clean from thinking evil. I can’t remember that last time I even thought about self-harm or suicide [Mimi, female, 16 years].*

##### “I won’t hang myself because of what somebody says”: use of substitutes

Some of the adolescents reported that they distract themselves from negative emotions related to events which could potentially trigger self-harm, by pursuing alternatives. Mostly, the alternatives are not intended to harm the self but are equally self-destructive at least in the long-term.

*Sometimes, where I live [slum name* 3*], some of the neighbours accuse me of things I haven’t done […] They tempt me a lot, but I keep [my] cool […] When it happens like that and I feel very angry, because I don’t want to fight anyone or beat up somebody or even do something to myself, I just leave the scene. I go out to our base to smoke [marijuana] and sit there for a while before I come back home, or sometimes I enter my room and take one or two tablets of tramol* 4*so that I can sleep off […] Because I don’t want all the false accusations to bother me or push me to do something bad to someone or to myself. I won’t hang myself because of what somebody says about me which is not true. So, the medicine [tramadol] helps a lot (Lewis, male, 15 years).*

## Discussion

The accounts of the adolescents we interviewed were elaborated more along the lines of social interactions with others, moral standards, and familial relationships, with little emphasis on individual level experiences such as emotional states and thoughts. While they reported both intrapersonal and interpersonal motives for their self-harm, they mostly externalised responsibility for their self-harm to circumstances and forces beyond their control.

Although the adolescent participants viewed self-harm as personally helpful in bringing release from emotional distress, they mostly evaluated their self-harm as socio-culturally undesirable and injurious to significant others; an evaluation that seemed to have motivated their non-repetition of self-harm. They relied on formal and informal support to stop or recover from self-harm. This pattern was especially a feature of the responses of street-connected adolescents - their overarching orientation for survival and sense of autonomy were implicated as key protective factors against self-harm [49].

The emphasis on family relationships, social roles, morals, and context-specific factors in the narratives of the adolescents is consistent with the construction of the self and meaning-making system in collectivistic societies such as Ghana [50–52]. In these societies, collective norms, community characteristics and other-centred social roles and interactions, rather than individual specific characteristics and experiences, constitute the frame of reference in the construction and presentation of the self and meaning-making in daily life [50, 53]. Based on this communal world view, the accounts of some of the adolescents in this study portray self-harm in adolescents as relational acts that can be interpreted as a protest against harsh punishment, abuse and powerlessness, assertion of innocence, cry for help, and an appeal [54–56].

In the Caribbean, Asian and African contexts, families have absolute control and power over their young members and women, particularly the social relationships and sexual behaviours of females; children and adolescents are considered immature to contribute to the decisions which affect their lives; and there are strict rules of obedience and respect [36, 57–60]. Although many countries across Africa are witnessing steady social changes through Westernisation, media, and formal classroom education, which emphasise the freedom to exercise fundamental human rights, independence, assertiveness and individuality, most families and societies within the continent are still deeply rooted in patriarchy with strict adherence to rules guiding traditional power relationships [36, 61, 62]. Young people occupy the base of the traditional power hierarchy, with no rights to complain or protest injustice [35, 36, 63]. Young people who break the rules of obedience and respect and social comportment are often punished to deter repetition, save the honour of the family and protect traditional power relationship. Sometimes, significant other adult relatives (including parents and adult siblings) go overboard to abuse young people physically and emotionally in exercising their right to punish young people for coming into conflict with the family’s honour [36].

Young people can feel trapped and powerless in these family circumstances, and may resort to self-harm or suicide as an escape, a protest, or as a socially intense way to communicate their displeasure, or for the family to find it legitimate to punish the significant other adults for the excesses in exercising their right to control and punishment [19, 22, 64–66]. Consistent with this evidence, some of the adolescents in the present study used self-harm in communicative terms as a protest against powerlessness, harsh punishment and abuse by their family, to assert their innocence of accusations of involvement in unapproved romantic relationships and immoral sexual behaviours, as a cry for help to escape material and financial difficulties, and as an appeal, requesting changes in the behaviours of their primary caregivers.

The adolescents’ post-hoc appraisal of their self-harm as socially and religiously immoral, influencing the likelihood of repetition, is not particularly surprising as the socialisation of children and young people in Ghana is mainly informed by religious beliefs and mores [34, 36]. What is however not clear is “how” these religious and interpersonal moralities influence the cessation or recovery from self-harm, nor indeed why such belief systems do not stop self-harm before it starts. It appears that, with the benefit of hindsight, some of the adolescents have learnt to appraise their self-harm retrospectively as religiously sinful and interpersonally injurious to others, and this replacement of thoughts appears to help the adolescents to avoid self-harming.

As expected, most of the adolescents who reported cessation or recovery from self-harm indicated that they also relied on informal support sources. Family support featured in the narratives of the in-school adolescents, whereas support from friends featured prominently in the accounts of the street-connected adolescents. This is consistent with evidence from one-time cross-sectional and longitudinal studies [67–70]. In Ghana, in-school adolescents typically live with and under the guardianship of their biological parents or kinship foster parents [36], making family relationship and support a critical component in the growth and welfare of adolescents [71]. Street-connected adolescents in Accra mostly live alone, hence tend to form social relationships and network of friends that serve not only as a source of support in times of difficulty but also as a survival strategy [49, 72–74].

Most participants in both groups of adolescents showed a disinclination to seek help for their self-harm from formal sources, even though many of them believed that talking to a trusted adult about emotional problems is helpful: teachers, counsellors, nurses, and social workers. Besides social stigma and the possibility of the social and legal proscription of self-harm in Ghana accounting for this lack of help-seeking, the evidence could also be reflecting the more general low level of help-seeking by adolescents from formal or professional sources reported in the global literature [75–78]. Given the importance of professional help-seeking in the prevention and recovery from self-harm [77, 78], future studies from Ghana could consider examining the experiential accounts of adolescents on the specific factors presenting as barriers and facilitators of professional help-seeking and how to improve the factors that facilitate adolescent help-seeking within the school and charity facility contexts.

Among the street-connected adolescents, their overarching orientation for survival and sense of autonomy were implicated as key protective factors against self-harm. The adoption of potentially self-destructive alternatives as coping strategies to distract from self-harm was also common among the street-connected adolescents. Other research from Ghana suggests that the preoccupation of street-connected children and youth with daily survival overrides all other needs [49, 72]. Although mostly unhealthy and fraught with risks, street-connected children and youth have a strong sense of resilience [79, 80]; they do various menial jobs, whilst others engage in criminal activities as a means of livelihood [49, 73, 81]. Compared to young people in school who live with their families, most street-connected children and youth live without adult supervision and control, making street living associated with some relative sense of freedom and autonomy. Street-connected adolescents are adultified, fending for themselves and taking the key decisions which affect their life [82]. The implication is that family factors related to adolescent self-harm such as parental control over adolescent social relationships, sexual behaviours and earnings from work, strict parental monitoring, punishment by parent, and parent-child conflict may not be strongly identified with self-harm among street-connected adolescents.

The use of equally self-destructive activities such as substance abuse could be explained in terms of the unrestricted access to and cheaper prices of these substances, coupled with the stimulating and mood elevation effects of drugs, alcohol and certain medicines (tramadol is common) available on the streets [83]. In providing support and intervention towards the cessation and recovery from self-harm among street-connected adolescents, street social workers should teach positive coping strategies and assess whether the adolescent has substituted their self-harm with a pleasurable but equally self-destructive behaviour.

### Strengths and limitations of study

This study represents the first attempt at simultaneously including both in-school and street-connected adolescents and selected key adult stakeholders in the same qualitative study on exploring the perceptions and meanings of adolescent self-harm within an underserved context. The strength of this approach is that the data provide an initial integrative picture of how self-harm in adolescents is variously perceived and interpreted by the different groups of participants. Our results can be applied to both the Ghanaian situation and the situation in other countries within sub-Saharan Africa since the knowledge and practices related to socio-cultural norms and value systems, family life, education, and street living are more similar than different within and across countries in sub-Saharan Africa [34].

More girls than boys agreed to interviews. Greater representation of boys would have broadened further the diversity of the experiences of self-harm shared by the adolescents. Maybe the face-to-face nature of the interview discouraged potential participants, particularly boy - all three adolescents who opted to be interviewed via telephone were boys. Future studies may consider arranging to interview participants through telephone, as this mode appears to offer some boys an increased sense of privacy.

As discussed earlier, the criminalised and socio-culturally proscribed status of self-harm in Ghana might have created the tendency for some participants to misrepresent their lives in guarded and socially desirable ways, engage in non-disclosure, lie and use other impression management strategies. With this mind, the reliability of the accounts of the participants in this study cannot be fully guaranteed. Even though attempts have been made throughout the analysis to explore the meanings of the results beyond the face value of the views of the participants, there is the need to accept the interpretations of the findings with caution.

Similarly, the adolescent participants had to recall their (last episode of) self-harm, which for some participants was up to four or 8 years prior to this study. This considerable time lag could potentially lead to forgetting, distorted memories of the circumstances leading up to their self-harm and their emotional experiences at the time or might have modified the interpretation of their self-harm [84, 85].

It has also been mentioned earlier that, comparatively, the interviews with the in-school adolescents were comprehensive and longer than those with the street-connected adolescents. The street-connected frequently dozed off or showed reduced concentration after the first 20 or 25 min of the interview, so obtaining comprehensive narratives and responses to key probes was difficult.

## Conclusions

Self-harm in adolescents is an issue of public concern in Ghana. The first-person accounts of adolescents in this study implicate familial relational problems and interpersonal difficulties as proximally leading to self-harm in adolescents. Self-harm in adolescents is interpreted as an understandable response, and as a strong communicative signal in response to powerlessness and family relationship difficulties. These findings, which suggest that an overly clinical model of self-harm will not be widely supported by those with personal experience, need to be taken into consideration in the planning of services in Ghana. They suggest that intervention needs to be located in schools or local facilities, with a strong orientation towards support for social problem-solving, building supportive social networks and reducing the stigma and isolation experienced by the young people we interviewed. In a separate report (manuscript in preparation) we discuss the views of our participants and of others in Ghana who are familiar with the problem, about what such services could do to prevent or respond to self-harm in the country.

## Data Availability

The datasets used and/or analysed during the current study are available from the corresponding author on reasonable request.

## Abbreviation

WHO: World Health Organization

## Footnotes

1 In Ghana, trotros are privately owned minibuses that travel fixed routes departing to their destinations when filled, and they can be boarded anywhere along the route. A trotro is typically operated by two people: a driver and a conductor (also called ‘trotro mate’ or ‘mate’). Usually, the driver employs the mate (who is typically a school drop-out, an unemployed youth, or a street-connected young person) and pays him after the day’s work.

2 “Kayayei” is the plural of “kayayoo”, a term used by the Ga people, the indigenous ethnic group in the Greater Accra region of Ghana, to refer to women or girls who engage in carrying goods for a fee. Etymologically, the term, kayayoo, is derived from two words, one from Hausa and one from the Ga language: “kaya” in Hausa means wares or goods, whilst “yoo” in the Ga language means woman or girl – the plural of “yoo” is “yei” in the Ga language. Thus, literally, “kayayei” translates “load-women” or “load-girls”. Usually, kayayei use large basins to carry goods and loads for shoppers, shopkeepers, and traders for a fee. Agarwal S, Attah M, Apt N, Grieco M, Kwakye EA, Turner J: **Bearing the weight: the kayayoo, Ghana’s working girl child**. *International Social Work* 1997, **40**(3):245–263. DOI: 10.1177/002087289704000302.

3 Name of slum anonymised for ethical reasons

4 Tramol is the informal term used in Ghana in reference to the painkiller tramadol. Non-medical use of tramadol is currently a challenge in Ghana, particularly, among young people – who are students, drivers, sex workers, street-dwelling, and even farmers. Access has been blamed mainly on smuggling and illicit distribution by untrained and unlicensed vendors in the open markets, who sell it at cheaper prices. Salm-Reifferscheidt L: **Tramadol: Africa’s opioid crisis**. *The Lancet* 2018, **391**(10134):1982–1983. DOI: 10.1016/s0140-6736(18)31073-0. Klein A: **Drug problem or medicrime? Distribution and use of falsified Tramadol medication in Egypt and West Africa**. *Journal of Illicit Economies and Development* 2019, **1**(1):52–62. DOI: 10.31389/jied.10.
